# Evaluation of the Use of the Polyubiquitin Genes, *Ubi4* and *Ubi10* as Reference Genes for Expression Studies in *Brachypodium distachyon*

**DOI:** 10.1371/journal.pone.0049372

**Published:** 2012-11-14

**Authors:** John P. Chambers, Ali Behpouri, Alison Bird, Carl K-Y. Ng

**Affiliations:** School of Biology and Environmental Science, University College Dublin, Belfield, Dublin, Ireland; University of Massachusetts Amherst, United States of America

## Abstract

**Background:**

*Brachypodium distachyon* is emerging as the model plant for temperate grass research and the genome of the community line Bd21 has been sequenced. Additionally, techniques have been developed for *Agrobacterium*-mediated transformation for the generation of T-DNA insertional lines. Recently, it was reported that expression of the polyubiquitin genes, *Ubi4* and *Ubi10* are stable in different tissues and growth hormone-treated plant samples, leading to the conclusion that both *Ubi4* and *Ubi10* are good reference genes for normalization of gene expression data using real-time, quantitative PCR (qPCR).

**Principal Findings:**

Mining of the Joint Genome Institute (JGI) 8X *Brachypodium distachyon* genome assembly showed that *Ubi4* and *Ubi10* share a high level of sequence identity (89%), and *in silico* analyses of the sequences of *Ubi4* (Bradi3g04730) and *Ubi10* (Bradi1g32860) showed that the primers used previously exhibit multiple binding sites within the coding sequences arising from the presence of tandem repeats of the coding regions. This can potentially result in over-estimation of steady-state levels of *Ubi4* and *Ubi10*. Additionally, due to the high level of sequence identity between both genes, primers used previously for amplification of *Ubi4* can bind to *Ubi10* and vice versa, resulting in the formation of non-specific amplification products.

**Conclusions:**

The results from this study indicate that the primers used previously were not sufficiently robust and specific. Additionally, their use would result in over-estimation of the steady-state expression levels of *Ubi4*. Our results question the validity of using the previously proposed primer sets for qPCR amplification of *Ubi4* and *Ubi10*. We demonstrate that primers designed to target the 3′-UTRs of *Ubi4* and *Ubi10* are better suited for real-time normalization of steady-state expression levels in *Brachypodium distachyon*.

## Introduction

*Brachypodium distachyon* is emerging as the model plant species for temperate grass research [1]. The *B. distachyon* genome has been sequenced using the community inbred line Bd21 [2] and the sequence data produced by the US Department of Energy Joint Genome Institute (http://www.jgi.doe.gov/) can be accessed online via the Brachypodium Genome Resource (http://www.brachypodium.org). In addition to a sequenced genome, *B. distachyon* is also amenable to *Agrobacterium*-transformation [2]–[4], thereby facilitating functional genomics studies through the generation of T-DNA insertional mutants that can be used for subsequent phenotypic characterization.

Recently, Hong *et al.* (2008) [5] reported that the steady-state expression levels of several genes are stable and are therefore useful for normalization of gene expression levels by real-time, quantitative PCR. These include *UBC18*, a gene encoding a ubiquitin-conjugating enzyme, *SamDC*, a gene encoding a S-adenosylmethionine decarboxylase, and 2 polyubiquitin genes, *Ubi4* and *Ubi10*. These genes were identified and primers were designed based on EST sequences. The authors reported that the steady-state expression levels of both *Ubi4* and *Ubi10* are stable in different tissues and growth hormone-treated plant samples, leading them to conclude that both *Ubi4* and *Ubi10* are good reference genes for normalization of gene expression data by qPCR [5].

Polyubiquitin genes are often characterized by tandem repeats of the coding regions and in general, the number of ubiquitin coding regions per gene varies from 3 to 6 [6]. In *Arabidopsis thaliana*, tandem repeats of the ubiquitin coding regions may not be exact copies and contain nucleotide variations leading to amino acid substitutions [6]. Comparison of the coding sequences of *Ubi4* and *Ubi10* obtained from the Joint Genome Institute (JGI) 8X *B. distachyon* genome assembly showed that these 2 genes exhibit a very high level of sequence identity (89%) and this was confirmed by independently cloning and sequencing the genes. Additionally, both *Ubi4* and *Ubi10* do not appear to contain introns in their coding regions, consistent with observations of the structure of polyubiquitin genes from *A. thaliana* [6]. We showed that the primers designed by Hong *et al.* (2008) [5] (Ubi4Fw, Ubi4Rv, Ubi10Fw, Ubi10Rv) exhibit multiple binding sites within the coding region that can result in multiple PCR amplification products. Interestingly the multiple banding was not detected by melt curve analysis but only by gel electrophoresis of the PCR products. Additionally, we demonstrate that using the primers, Ubi4Fw, Ubi4Rv, Ubi10Fw, and Ubi10Rv, for qPCR can result in over-estimation of steady-state levels of *Ubi4*. This problem is exacerbated by the observation that these primers can also recognize *Ubi10* transcripts as templates, resulting in the formation of non-specfic amplification products. We further show that primers designed to amplify *Ubi10* primers may compete for the same binding sites and this may influence the efficiency of the PCR reaction. The *Ubi10* primers also show some weak affinity for the *Ubi4* gene sequence.

The results from this study indicate that the primers, Ubi4Fw, Ubi4Rv, Ubi10Fw, Ubi10Rv [5] were not sufficiently robust and specific. It is important to note that these primers were designed from EST sequences as the full *B. distachyon* was not available at that time. Our results show that these primers, Ubi4Fw, Ubi4Rv, Ubi10Fw, and Ubi10Rv, are not suitable for amplification of *Ubi4* and *Ubi10*, and demonstrate that primers designed to target the 3′-UTRs of *Ubi4* and *Ubi10* are better suited for real-time normalization of steady-state expression levels in *B. distachyon*.

Another finding of importance from this investigation is the apparent inability of qPCR melt curve analysis to pick up the multiple banding detected by gel electrophoresis of the products and this can be attributed to the presence of tandem repeats within the coding sequence. These findings further re-enforces the need for careful and precise planning and testing of experimental setups for qPCR experiments.

## Results

### Reverse Transcriptase PCR of Reference Genes *Ubc18*, *Ubi4*, and *Ubi10* from 2-week Old Leaves of *B. distachyon*

**Figure 1:**
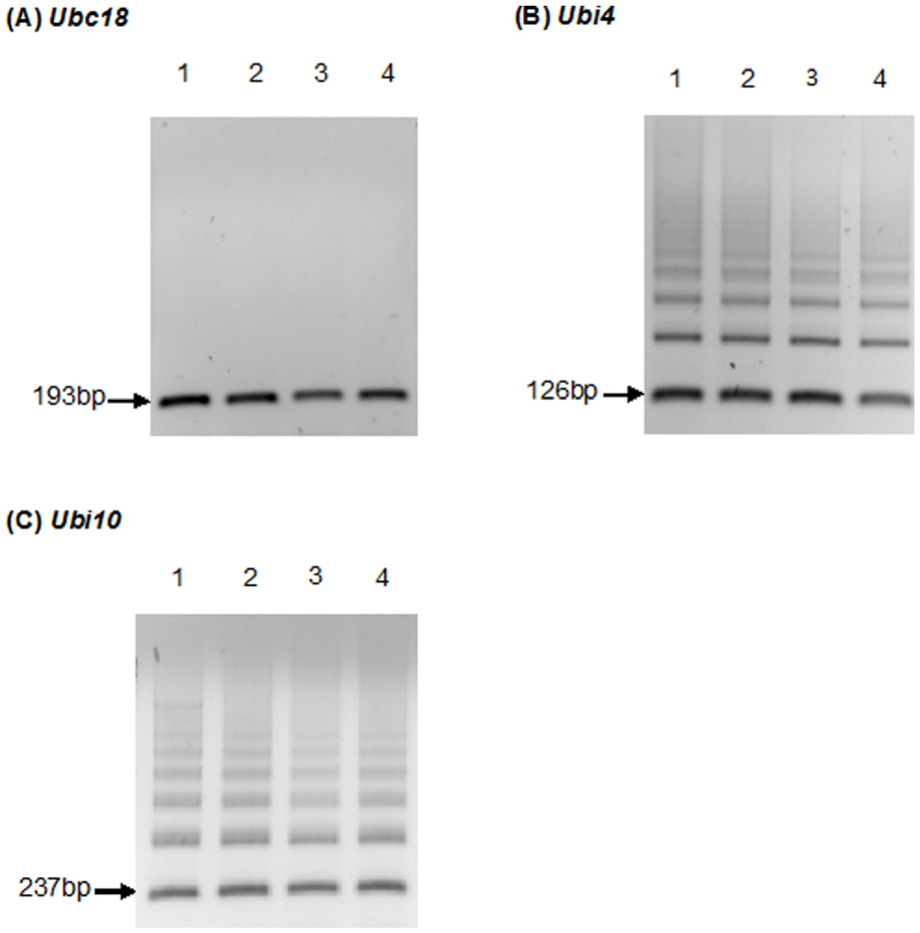
Gel electrophoresis of products from PCR amplification of the housekeeping genes, *Ubc18*, *Ubi4*, and *Ubi10* from cDNA derived from RNA isolated from 2-week old leaves. Lanes 1, 2, 3 and 4 in (A), (B) and (C) show the products of PCR amplification of cDNA templates from 4 individual plants. The expected sizes of the PCR products are 193 bp (*Ubc18*), 126 bp (*Ubi4*), and 237 bp (*Ubi10*). The results shown are reverse gel images (dark bands on white backgrounds) from 25 amplification cycles. Gel images are representative of 3 independent experiments.

Complementary DNAs (cDNAs) were used as template for PCR amplification with the appropriate primers (Ubi4Fw, Ubi4Rv, Ubi10Fw, and Ubi10Rv) [5]. Following RT-PCR amplification, gel electrophoresis was used to visualize the size of the PCR products to determine if products of the expected sizes were obtained (Figure 1). The expected sizes of the PCR products are 193 bp (*Ubc18*), 126 bp (*Ubi4*), and 237 bp (*Ubi10*). Figure 1A shows the results of PCR amplification of the *Ubc18* gene. It can be seen that this PCR reaction yielded a single band of the expected size. In contrast, the PCR amplification of *Ubi4* (Figure 1 B) and *Ubi10* (Figure 1 C) yielded multiple bands. To ensure that the multiple banding patterns observed for *Ubi4* (Figure 1 B) and *Ubi10* (Figure 1 C) were not due to variations in amplification specificities, a gradient PCR using temperature ranging from 55°C to 65°C was performed, and multiple products were obtained regardless of the annealing temperatures tested (Figure S1). Additionally, the multiple banding patterns observed were not due to genomic contamination as no amplification was observed in no reverse transcriptase (RT) controls (Figure S1).

### *In silico* Analysis of *Ubi4* and *Ubi10*, and Primer Binding Sites within the Coding Sequences

**Figure 2:**
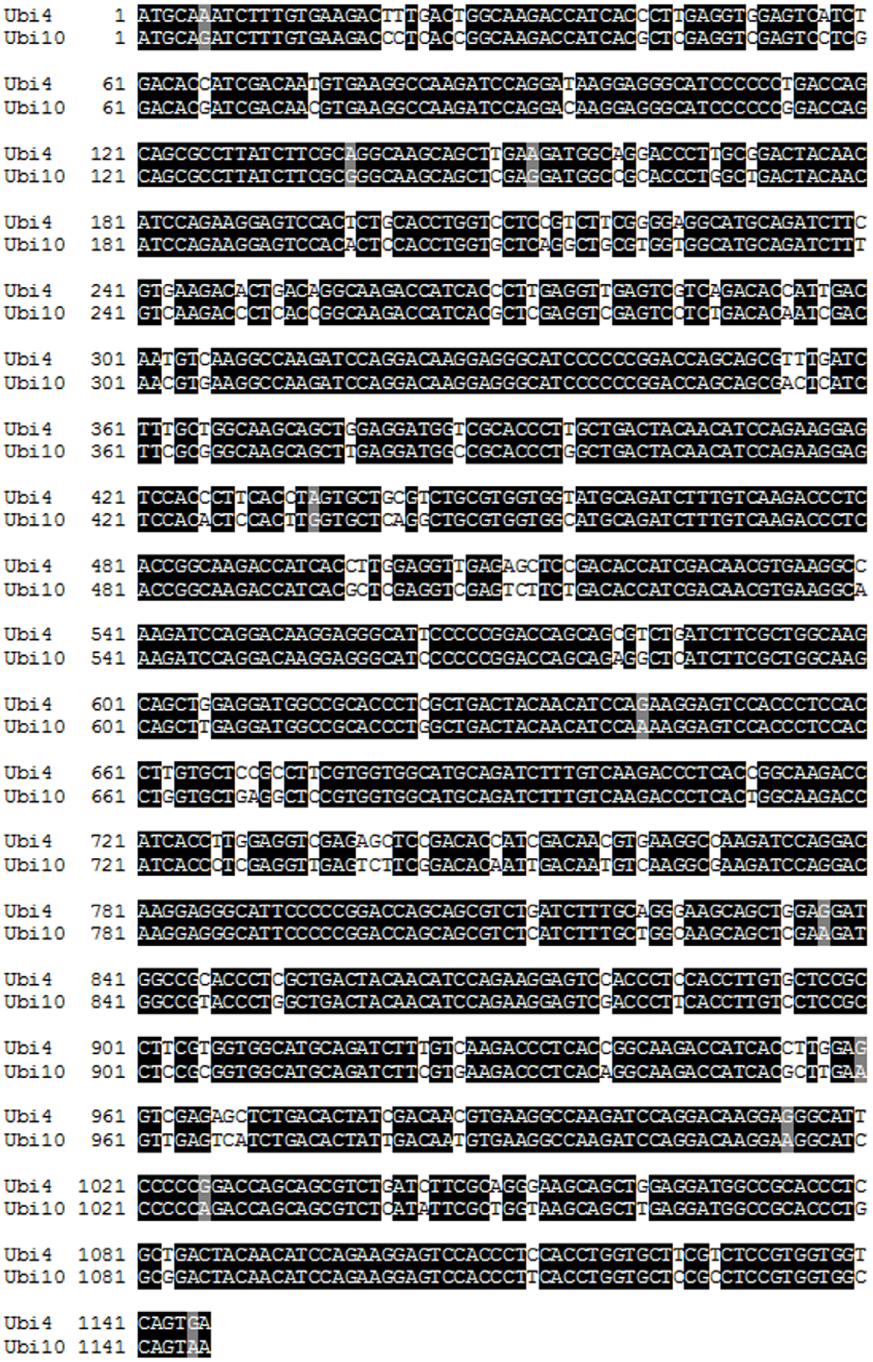
CLUSTAL W alignment of *Ubi4* and *Ubi10* sequences. Alignment of the nucleotide sequences of *Ubi4* (Bradi3g04730) and *Ubi10* (Bradi1g32860) showed a very high level of sequence identity (89%; BLASTN 2.2.22) between the two polyubiquitin genes. Identical nucleotides are shown in black and nucleotides shaded in grey refer to conserved purines using the Boxshade program.

In order to understand the multiple banding associated with RT-PCR of *Ubi4* and *Ubi10*, we mined the JGI 8X *B. distachyon* genome assemblies via BrachyBase (http://www.brachybase.org) to obtain the coding sequences of *Ubi4* and *Ubi10* and compared both sequences using BLASTN (version 2.2.22). Our analyses showed that both genes exhibit a very high level (89%) of sequence identity (Figure 2). The primers designed by Hong *et al.* (2008) [5] show either perfect matches with the coding sequences (*Ubi4* and *Ubi10*) or 1 or 2 base mismatches (Figures S2, S3). This can potentially result in over-estimation of the steady-state levels of *Ubi4* and *Ubi10* and may account for the observation of multiple products arising from PCR amplification.

### Reverse Transcriptase (RT)-PCR Amplification of *Ubi4* and *Ubi10*

**Figure 3:**
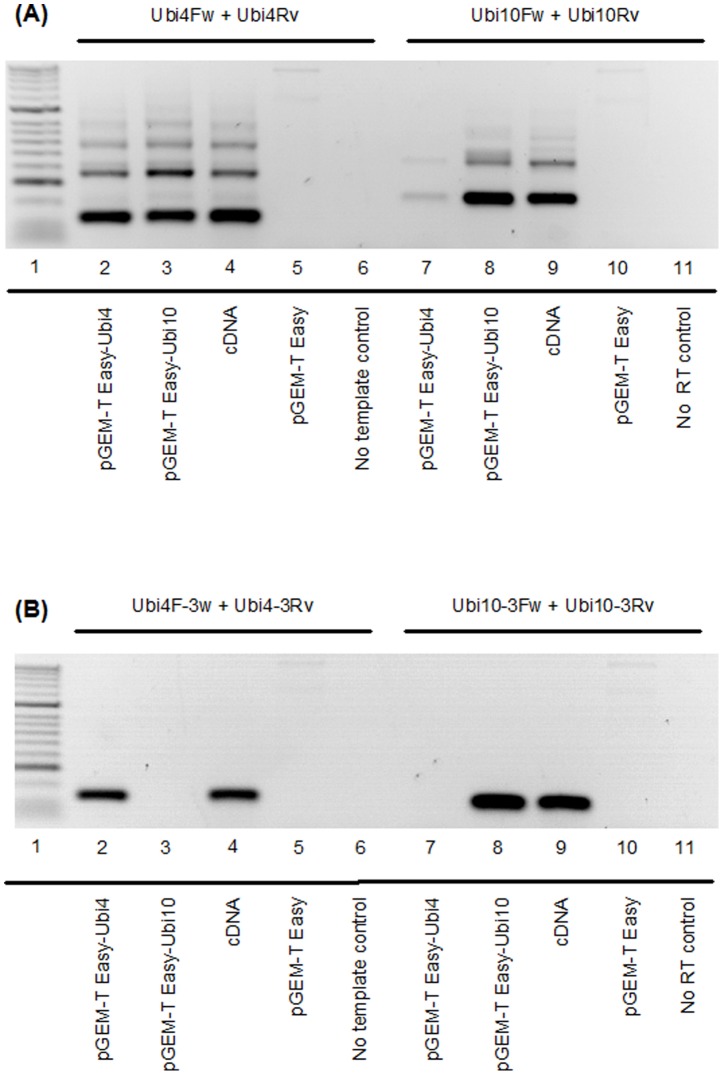
PCR amplification of *Ubi4* and *Ubi10* from various templates. (A) Primers used for amplification of *Ubi4* (Ubi4Fw and Ubi4Rv) and *Ubi10* (Ubi10Fw and Ubi10Rv) are according to Hong *et al.* (2008) [5]. Lane 1: molecular weight marker (Bioline HyperLadder 1). The templates used are indicated for each lane. Empty vector (pGEM-T Easy) and no RT reactions were included as controls. The results shown are reverse gel images (dark bands on white backgrounds) from 25 amplification cycles. (B) Primers targeting the 3′-UTRs of *Ubi4* (Ubi4-3Fw and Ubi4-3Rv) and *Ubi10* (Ubi10-3Fw and Ubi10-3Rv) were used for amplification of the various templates. Lane assignments and templates used as in (A). Gel images are representative of 3 independent experiments.

To validate the results obtained from RT-PCR of cDNA (Figure 1) and *in silico* analyses (Figure 2; Figures S2 and S3), we reverse transcribed cDNA from total RNA isolated from *B. distachyon* Bd21 to clone the cDNAs corresponding to *Ubi4* and *Ubi10*. Due to the high level of sequence identity between *Ubi4* and *Ubi10* (Figure 2), we designed primers to bind to the predicted 5′- and 3′-untranslated regions (UTRs) in order to enable us to clone the full-length coding sequence of both *Ubi4* and *Ubi10*. The corresponding cDNAs were then sub-cloned into pGEM T-Easy and the sequences verified. To determine the cross-reactivity of the primers, we used the plasmid clones as templates for PCR. Using purified plasmids (pGEM T-Easy) containing the coding sequence of *Ubi4*, we were able to demonstrate that the *Ubi4* primers (Ubi4Fw, Ubi4Rv) were indeed able to bind to multiple sites within the coding sequence (as predicted by *in silico* analysis), leading to the formation of multiple PCR amplification products (Figure 3 A). The multiple banding patterns observed are not due to genomic contamination as no bands were detected in the no RT controls (Figures 3 A, B). Additionally, we were able to demonstrate that *Ubi10* primers (Ubi10Fw, Ubi10Rv) were able to bind to *Ubi4*, resulting in the formation of non-specific PCR amplification products (Figure 3 A).

We also used purified plasmids (pGEM T-Easy) containing the coding sequence of *Ubi10* to demonstrate that *Ubi10* primers (Ubi10Fw, Ubi10Rv) were able to bind to multiple sites within the coding sequence, resulting in the formation of multiple PCR amplification products (Figure 3 A) and that primers used for amplification of *Ubi4* (Ubi4Fw, Ubi4Rv) also resulted in the formation of non-specific PCR amplification products when *Ubi10* was used as the template (Figure 3 A).

To circumvent the problem associated with the formation of non-specific amplification products and over-estimation of steady-state levels of *Ubi4* and *Ubi10* transcripts using primers Ubi4Fw, Ubi4Rv, Ubi10Fw, and Ubi10Rv, we designed primers targeting the 3′-UTR of *Ubi4* and *Ubi10*. RT-PCR amplification using primers targeting the 3′-UTRs of *Ubi4* (Ubi4-3Fw, Ubi4-3Rv) and *Ubi10* (Ubi10-3Fw, Ubi10-3Rv) clearly demonstrated the formation of one specific amplification product corresponding to 155 bp and 138 bp for *Ubi4* and *Ubi10*, respectively (Figure 3 B).

### Comparative qPCR of *Ubi4* and *Ubi10* using Different Primer Sets to Determine Amplification Specificity

To ascertain the effects of non-specific primer binding on the levels of steady-state transcripts, real-time quantitative PCR (qPCR) was performed using Ubi4Fw, Ubi4Rv; Ubi10Fw, and Ubi10Rv, and primers designed to target the 3′-UTRs (Ubi4-3Fw, Ubi4-3Rv; Ubi10-3Fw, and Ubi10-3Rv). In line with Kapa Sybr Green protocols, various primer concentrations were tested for the four primer sets at an annealing temperature of 60°C. We observed that primer concentrations of 100 nM resulted in the most efficient amplification (data not shown). Using plasmids containing either *Ubi4* or *Ubi10* as template, serial dilution series qPCRs were run in order to calculate primer efficiencies. PCR efficiency (E) values and r^2^ values were calculated for each run in order to compare primer sets. The primer set Ubi4-3Fw and Ubi4-3Rv showed an E value of 102% and an r^2^ value of 0.999 whereas the primer set Ubi4Fw and Ubi4Rv showed an E value of 95% and r^2^ value of 0.999. The primer set Ubi10-3Fw and Ubi10-3Rv showed an E value of 111% and an r^2^ value of 0.983 while the primer combination of Ubi10Fw and Ubi10Rv showed an E value of 117% and r^2^ value of 0.986.

**Figure 4:**
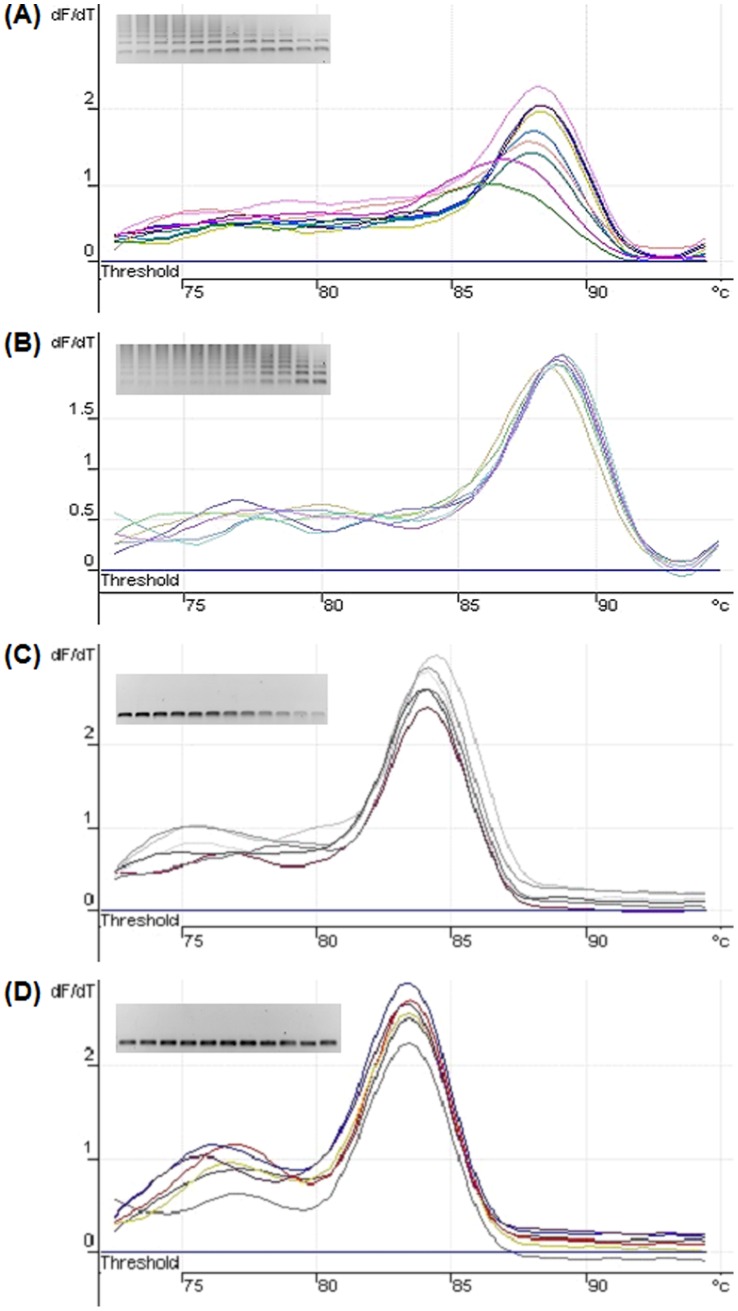
Comparative dissociation curve analyses of qPCR amplification from various primer combinations. (A) Dissociation curve data for primer combination Ubi4Fw and Ubi4Rv. Inset: Gel electrophoresis of qPCR amplification products showing the presence of multiple PCR products. Reverse gel image (dark bands on white background). (B) Dissociation curve data for primer combination Ubi10Fw and Ubi10Rv. Inset: Gel electrophoresis of qPCR amplification products showing the presence of multiple PCR products. Reverse gel image (dark bands on white background). (C) Dissociation curve data for primer combination Ubi4-3Fw and Ubi4-3Rv. Inset: Gel electrophoresis of qPCR amplification products showing the presence of one PCR product. Reverse gel image (dark bands on white background). (D) Dissociation curve data for primer combination Ubi10-3Fw and Ubi10-3Rv. Inset: Gel electrophoresis of qPCR amplification products showing the presence of one PCR product. Reverse gel image (dark bands on white background).

Interestingly, all primer pair combinations gave a single curve when dissociation analysis was carried out on the products of qPCR (Figures 4 A to D). However, gel electrophoresis of the amplification reactions showed that Ubi4Fw and Ubi4Rv and Ubi10Fw and Ubi10Rv primer combinations resulted in multiple PCR products (Figures 4 A, B) whereas primer combinations, Ubi4-3Fw and Ubi4-3Rv, and Ubi10-3Fw and Ubi10-3Rv (amplification of 3′-UTRs) resulted in only one PCR amplification product (Figures 4 C, D).

**Figure 5:**
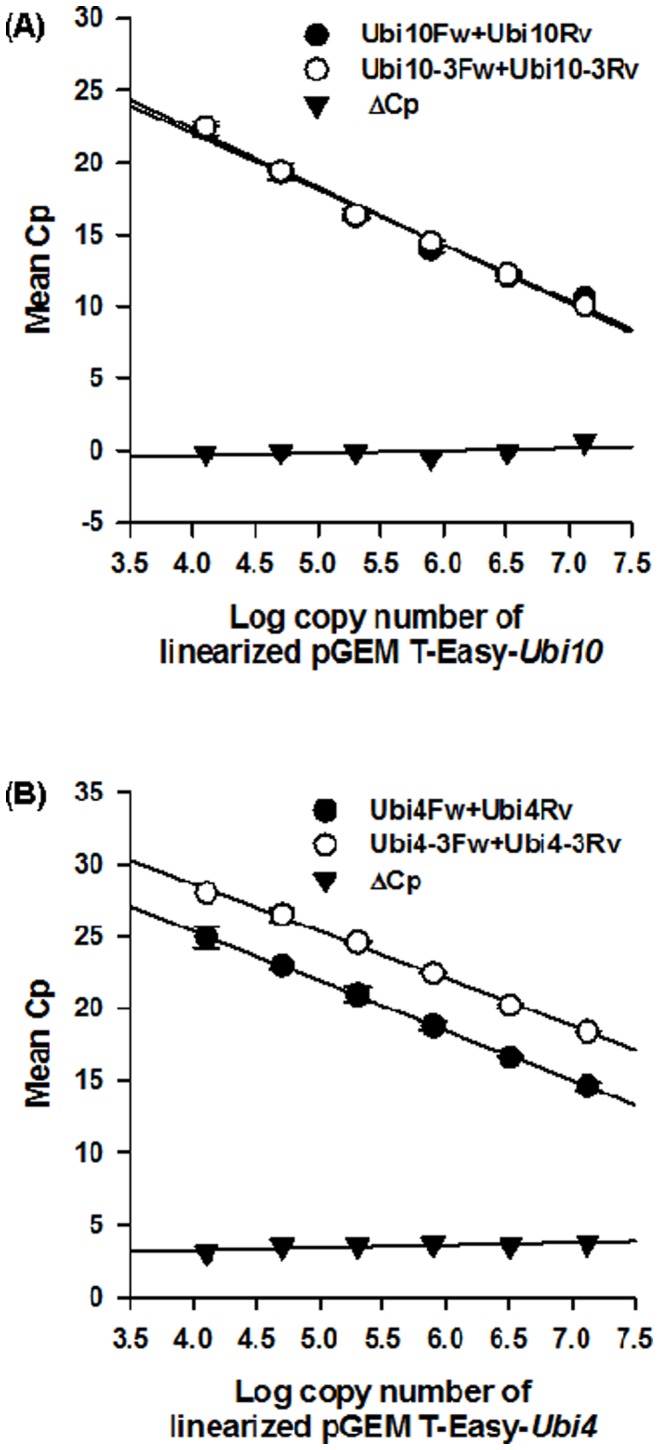
Real time PCR standard curves for the primer sets used. (A) Standard curve generated using a serial dilution of pGEM T-Easy-*Ubi10* as template. The Cp values obtained at each concentration for Ubi10Fw and Ubi10Rv [5] are shown as white circles. The Cp values obtained at each concentration for Ubi10-3Fw and Ubi10-3Rv are shown as black circles. The ΔCp between both sets of primers at each concentration is indicated by triangle. Error bars are shown for each point. (B) Standard curve generated using a serial dilution of pGEM T-Easy-*Ubi4* as template. The Cp values obtained at each concentration for Ubi4Fw and Ubi4Rv [5] are shown as white circles. The Cp values obtained at each concentration for Ubi4-3Fw and Ubi4-3Rv are shown as black circles. The ΔCp between both sets of primers at each concentration is indicated by triangle. Values are mean ± SE (n = 3).

From Figure 3A it can be seen that the primer combination Ubi4Fw and Ubi4Rv exhibit strong cross reactivity with *Ubi10* as a template whereas Ubi10Fw and Ubi10Rv showed only weak cross reactivity with *Ubi4* as a template. This suggests the possibility of overestimation of steady-state transcript levels during qPCR when using Ubi4Fw and Ubi4Rv primers. To verify this, we used purified *Ubi4* and *Ubi10* templates (linearized pGEM T-Easy plasmids containing *Ubi4* or *Ubi10*) for qPCR. Using a dilution series of linearized pGEM T-Easy-*Ubi10* template, comparable Cp values were obtained for both primers sets Ubi10Fw and Ubi10Rv and Ubi10-3Fw and Ubi10-3Rv (Figure 5 A). In contrast, using a dilution series of linearized pGEM T-Easy-*Ubi4* template, a difference in Cp (ΔCp) value of *ca.* 3 was observed between the 2 different primer sets, with the primers Ubi4Fw and Ubi4Rv consistently exhibiting a lower Cp value compared to primers Ubi4-3Fw and Ubi4-3Rv (Figure 5 B). This suggests that the primer set Ubi4Fw and Ubi4Rv, is likely to over-estimate the steady-state levels of *Ubi4* transcripts.

**Figure 6:**
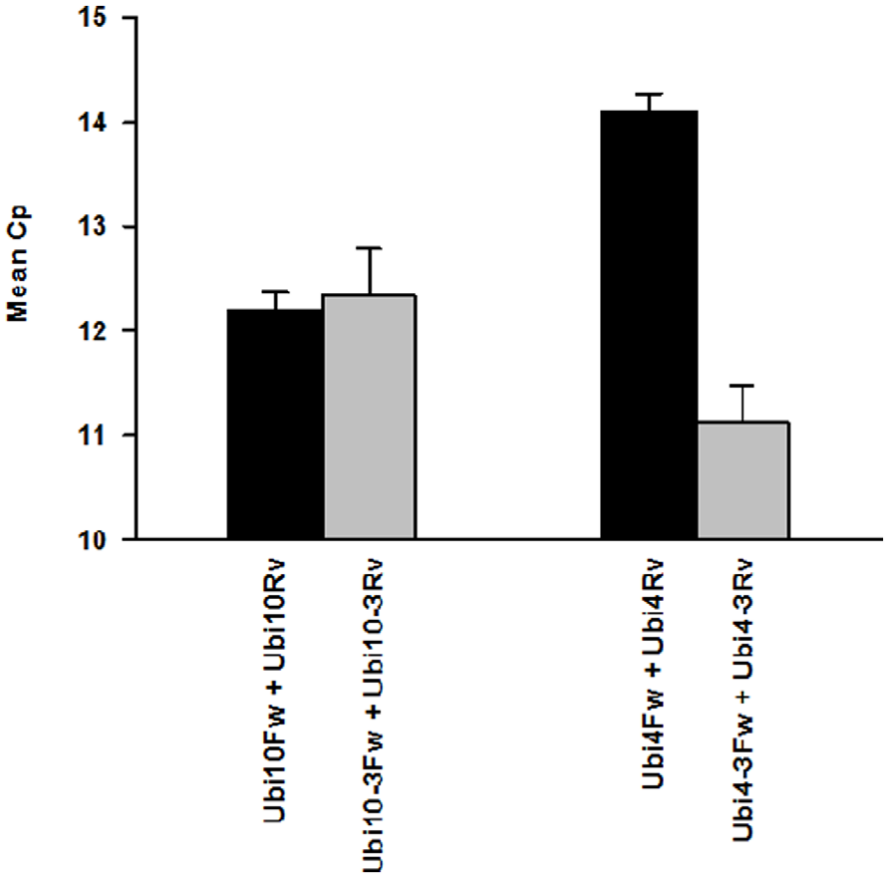
qPCR of *B. distachyon* cDNA using various primer combinations. Mean Cp observed when using cDNA as a template for each of the primer pairs, Ubi4 Fw and Ubi4Rv, Ubi10 Fw and Ubi10Rv, Ubi4-3Fw and Ubi4-3Rv, Ubi10-3Fw and Ubi10-3Rv. Values are mean ± SE (n = 3).

In order to ascertain if the results observed using linearized plasmids containing *Ubi4* and *Ubi10* as templates were reproducible for the analysis of transcript levels, qPCR was carried out using cDNA as template (Figure 6). Comparable mean Cp values were obtained for the 2 primer sets used for amplification of *Ubi10* (Figure 6). In contrast, our results clearly showed that there is a significant difference in the mean Cp value between primer sets used for amplification of *Ubi4* with primer combination Ubi4Fw and Ubi4Rv showing a lower mean Cp value compared to primer set Ubi4-3Fw and Ubi4-3Rv (Figure 6). The ΔCp value of 3 between these 2 primer sets (Figure 6) are consistent with results obtained using linearized plasmids containing *Ubi4* (Figure 5 B).

### Suitability of Primer Sets Designed to Amplify the 3′-UTRs for use in Developmental Studies

**Figure 7:**
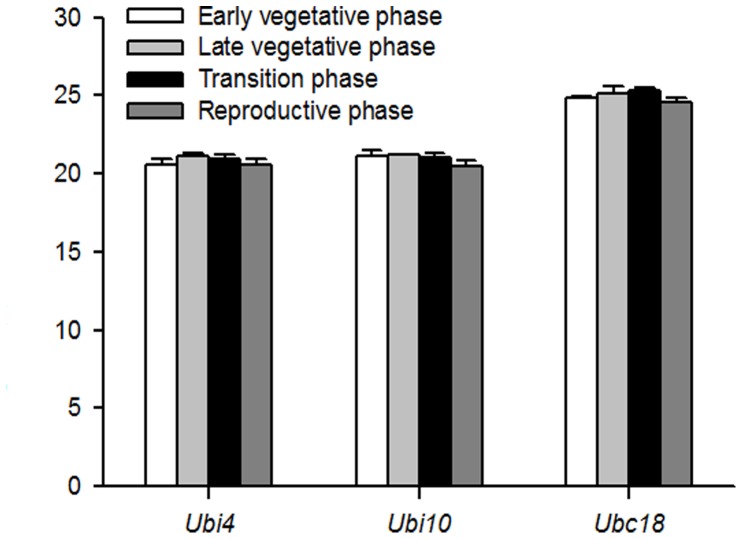
qPCR determination of *Ubi4*, *Ubi10* and *Ubc18* expression during development in *B. distachyon*. cDNA from 4 different developmental stages were used: early vegetative stage, late vegetative phase, transition phase, and reproductive phase. Primers designed to target the 3′-UTRs (Ubi4-3Fw and Ubi4-3Rv, Ubi10-3Fw and Ubi10-3Rv) were used for amplification of *Ubi4* and *Ubi10*. For amplification of *Ubc18*, primers used were those reported by Hong *et al.* [5]. The mean Cp values are average of 3 biological replicates ± SE (n = 3). Three technical replicates were conducted for each biological replicate.

To determine the suitability of the new primer sets (Ubi4-3Fw and Ubi4-3Rv, and Ubi10-3Fw and Ubi10-3Rv) for normalization in developmental studies, we isolated RNA from *B. distachyon* plants at various developmental stages according to Hong *et al.* [5]: Early vegetative phase, late vegetative phase, transition phase, and reproductive phase. Our results show that the mean Cp values for *Ubi4* and *Ubi10* (Figure 7) are not significantly different at the different developmental stages, indicating that these 2 genes are stably expressed and suitable for use as reference genes for developmental studies. Additionally, we also show that *Ubc18*, which Hong *et al.* [5] has identified as a good reference gene is also stably expressed during development (Figure 7).

## Discussion

Real time, quantitative PCR (qPCR) is a powerful technique for quantifying steady-state transcript levels. Furthermore, several groups have reported that qPCR may be used to ascertain transgene copy numbers in mutants [7]–[8]. Hong *et al.* (2008) [5] proposed 3 genes as suitable reference genes for normalization of gene expression under various experimental conditions. Of the three genes tested, *Ubc18* was shown to be most suitable for comparison between plant samples and between different stages of development, while *Ubi4* and *Ubi10* were demonstrated to be suitable for comparisons between different plant tissues [5].

We showed that using the primer pairs designed by Hong *et al.* (2008) [5] for amplification of *Ubi4* and *Ubi10* resulted in the formation of multiple PCR products (Figure 1 B, C), indicating non-specific priming of the target templates. Attempts to optimize annealing temperature from 55°C to 65°C (the theoretical melting point of the primers) did not result in any increase in amplification specificity (Figure S1). We cloned *Ubi4* and *Ubi10* and *in silico* analyses of the sequences indicated that both genes share an 89% sequence identity (Figure 2), and revealed potential multiple binding sites for both Ubi10Fw, Ubi10Rv and Ubi4Fw, Ubi10Rv primers in both *Ubi4* and *Ubi10* (Figures S2 and S3). We showed that the 3′-UTRs of both *Ubi4* and *Ubi10* are the only regions that allow primers to be designed that would not result in cross reactivity or produce non-specific PCR products (Figure S4). Primers were designed for amplification from the 3′-UTRs of *Ubi4* (Ubi4-3Fw, Ubi4-3Rv) and *Ubi10* (Ubi10-3Fw, Ubi10-3Rv), and all primer sets were tested on plasmids harbouring *Ubi10* and *Ubi4* (pGEM-T-Easy-*Ubi10* and pGEM-T-Easy-*Ubi4*) and cDNA, and the results clearly demonstrated the specificity of the primers designed to target amplification from the 3′-UTRs (Figures 3 A, B).

The ability of Ubi4Fw and Ubi4Rv, and Ubi10Fw and Ubi10Rv to cross react with both *Ubi4* and *Ubi10* and produce multiple PCR products suggest a problem with the primer design. Interestingly, a single melting peak (often used as a test of amplification specificity of qPCR) was obtained for all primer sets tested although multiple PCR products were obtained only when primers Ubi4Fw, Ubi4Rv, Ubi10Fw, and Ubi10Rv, were used (Figure 4). This can be attributed to the formation of multiple products of similar melting temperatures due to the presence of tandem repeats within the coding sequences of *Ubi4* and *Ubi10*.

As multiple PCR products are formed when the primers, Ubi4Fw, Ubi4Rv, Ubi10Fw, and Ubi10Rv, were used, it suggests that the steady-state levels may be overestimated. Indeed, we observed that qPCR using Ubi4Fw and Ubi4Rv primers consistently yielded a lower mean Cp value of 3 when compared with qPCR using Ubi4-3Fw and Ubi4-3Rv, indicating that the steady-state levels of *Ubi4* were over-estimated by 3-fold (Figure 5 B). This is consistent with the observation of 3 potential binding sites within the coding sequence of *Ubi4* for Ubi4Fw and Ubi4Rv as opposed to only one binding site for Ubi4-3Fw and Ubi4-3Rv on the 3′-UTR (Figure S2).

Interestingly, while multiple PCR products were also obtained using Ubi10Fw and Ubi10Rv, no differences in the mean Cp values were observed between primer sets Ubi10Fw, Ubi10Rv and Ubi10-3Fw, Ubi10-3Rv (Figure 5 A). This can be attributed to the sequence overlap between Ubi10Fw and Ubi10Rv (Figure S3) where binding of either the forward or reverse primer would exclude the other.

We show, using the primers designed to target the 3′-UTRs of *Ubi4* and *Ubi10* that both these genes are stably expressed under different developmental stages (Figure 6). Additionally, the expression of *Ubc18* is also stable during development (Figure 6). Together, this data set show that *Ubi4*, *Ubi10*, and *Ubc18*, can be use as reference genes for developmental studies.

Given the cross reactivity of the Ubi4Fw, Ubi4Rv and Ubi10Fw, Ubi10Rv primers, the results obtained for steady-state levels reported by Hong *et al.* (2008) [5], are questionable. Our results clearly highlight the need to empirically and comprehensively test all primers and PCR conditions to determine the suitability of reference genes for normalization of gene expression.

### Conclusions

The results from this study indicate that the primers designed by Hong *et al.* (2008) [5] were not sufficiently robust and specific. Additionally, their use would result in over-estimation of the steady-state levels of *Ubi4*. Our results question the validity of using the primer sets, Ubi4Fw, Ubi4Rv, Ubi10Fw, and Ubi10Rv, for amplification of *Ubi4* and *Ubi10* as reference genes for normalization of gene expression levels by qPCR in *B. distachyon*. However, it is important to note that the primer sequences were based on ESTs when the full *B. distachyon* information was not available. We demonstrate that primers designed to target the 3′-UTRs of *Ubi4* and *Ubi10* are better suited for normalization of steady-state transcript levels by qPCR in *B. distachyon*, and we demonstrated the suitability of *Ubi4*, *Ubi10*, and *Ubc18* as reference genes for developmental studies. We also highlight a potential limitation of using melt curve analysis as the sole determinant of amplification specificity and demonstrate the absolute requirement for gel electrophoresis of PCR products to support melt curve analyses.

## Materials and Methods

### Plant Growth

Mature seeds of *Brachypodium distachyon* Bd21 (kindly provided by Dr. David F. Garvin, USDA-ARS, MN, USA) were soaked in distilled water for 2 hours before the upper and lower glumes were removed. Seeds were then soaked in 20% household bleach (∼ 1% sodium hypochlorite) for 3 min and washed 3 times with sterile distilled water. They were then placed on moist sterile filter paper in Petri dishes (9 cm Ø) at a density of 20 seeds per plate. Plates were stratified at 4°C for 3d in the dark before being transferred to a controlled temperature chamber (Sanyo, http://www.sanyo-biomedical.co.uk) at 25°C for 4d in the dark. Germinated seeds were then transferred to a compost:vermiculite (2∶1, v/v) mix (Shamrock multipurpose compost, Ireland) in 5×5 cm pots at a density of 1 seedling per pot, and placed in a Microlima 1750 climate controlled growth chamber (Snijders, http://www.snijders-scientific.nl/) under the following conditions: 16/8h and 24/18°C light/dark; PPFD of 250 µmol m^−2^s^−1^; relative humidity of 70%. Plants were watered daily.

### RNA Isolation and Cloning of *Ubi4* and *Ubi10*

Leaves from various stages of development of *B. distachyon* plants (as identified by Hong *et al* (2008) [5] were harvested and flash frozen in liquid nitrogen. The different stages are: early vegetative phase, late vegetative phase, transition phase, and reproductive phase. Total RNA was extracted from the leaves harvested from 4 plants with each plant representing one replicate using the RNeasy kit (Qiagen, http://www1.qiagen.com/) according to the manufacturer’s instructions. The quality and quantity of the total RNA was determined using a NanoDrop 1000 spectrophotometer (Thermo Scientific, http://www.nanodrop.com). 1000 ng of total RNA was treated with 1 U of DNaseI (Invitrogen, http://www.invitrogen.com/site/us/en/home.html) before being used for cDNA synthesis using 200 U of M-MLV Reverse Transcriptase (Invitrogen, http://www.invitrogen.com/site/us/en/home.html). The resultant cDNA was used for PCR amplication of *Ubi4* and *Ubi10* using KOD Hot Start DNA Polymerase (Novagen, http://www.merck-chemicals.com/life-science-research) using primers designed to the predicted 5′- and 3′-UTRs.

Ubi4utrFw: 5′-AGGCAATCTCGTCTTCTCCAATCG-3′.

Ubi4utrRv: 5′-ACCCAGGTATAGCAGCAGTTCCAA-3′.

Ubi10utrFw: 5′-CCAAACTCTCAATCGCACCGAGAA-3′.

Ubi10utrRv: 5′-ACACCCTGAACCAGACTTGTGAAC-3′.

The resulting PCR products were then sub-cloned into pGEM T-Easy (Promega, http://www.promega.com), and transformed into *E. coli* DH5α, and the plasmids isolated and purified and sequence verification of the full-length clones of *Ubi4* and *Ubi10* were conducted by Eurofins MWG Operon (http://www.the-mwg.com).

### Reverse Transcriptase-PCR (RT-PCR)

RT-PCR was conducted using 0.5 U of Go-Taq™ DNA polymerase (Promega, http://www.promega.com) and cDNA or purified plasmids as templates. The following cycling protocol was used for RT-PCR. Initial step (95°C for 3 min), 25 cycles (95°C for 1 min, 60°C for 1 min and 72°C for 1min), final step (72°C for 10 min). The sequences of the primers used (Ubi4Fv, Ubi4Rv, Ubi10Fv, and Ubi10Rv) were according to Hong *et al.* (2008) [5]. Primers were also designed to target the 3′-UTRs of *Ubi4* and *Ubi10* (Ubi4-3Fv, Ubi4-3Rv, Ubi10-3Fv, and Ubi10-3Rv). All primers were synthesized by Eurofins MWG Operon (Germany).

Ubi4Fv: 5′-TGACACCATCGACAACGTGA-3′.

Ubi4Rv: 5′-GAGGGTGGACTCCTTCTGGA-3′.

Ubi10Fv: 5′-TCCACACTCCACTTGGTGCT-3′.

Ubi10Rv: 5′-GAGGGTGGACTCCTTTTGGA-3′.

5′-GCTGTTGGAACTGCTGCTATACCT-3′.

5′-TTGCACCAAACCAACACACACCAG-3′.

5′-TGGACTTGCTTCTGTCTGGGTTCA-3′.

Ubi10-3Rv: 5′-TGGTACACAGGCATAACACTGACG-3′.

Note: Ubi4Rv and Ubi10Rv differ by only one base mismatch.

Ubi4Rv: 5′-GAGGGTGGACTCCTTCTGGA-3′.

Ubi10Rv: 5′-GAGGGTGGACTCCTTTTGGA-3′.

### Sub Cloning of *Ubi4* and *Ubi10* Genes for Sequencing

The pGEM®-T Easy Vector System (Promega, UK) was used for sub-cloning *Ubi4* and *Ubi10*. The sequences of *Ubi4* and *Ubi10* sub-cloned into pGEM T-Easy were verified by MWG Eurofins Operon sequencing lab (Germany).

### Real-time, Quantitative PCR (qPCR) of *Ubi4* and *Ubi10*

qPCR of steady state levels of *Ubi4* and *Ubi10* were conducted using 1.5 µl of cDNA (total RNA concentration of 75 ng) or 25 ng µl^−1^ of linearized pGEM-T-Easy plasmid containgin *Ubi4* or *Ubi10*. These were then serially diluted with H_2_O. A standard curve for each set of primers was carried out using a dilution series of 6 concentrations of linearized plasmid, and best fit line was obtained. From this the efficiency (E) of each set of primers was ascertained.

qPCR was carried out using Kapa SYBR® fast qPCR Universal Kit (Anachem, UK). Each reaction was made up to 20 µl, with 10 µl Kapa SYBR green master mix, 100 nM of forward and reverse primers and either 1.5 µl of cDNA or 1 µl of plasmid as starting template with 7.5 or 8 µl respectively, of distilled RNAase free H_2_O. 4 sets of primers were used for each template, Ubi4Fw and Ubi4Rv (Hong et al., 2008; [5]), Ubi10Fw and Ubi10 Rv (Hong et al. 2008; [5]), Ubi4-3Fw and Ubi4-3Rv (designed to target the 3′-UTR), and Ubi10-3Fw and Ubi10-3Rv (designed to target the 3′-UTR). Reactions were run in triplicate for each template and primer set on a RotorGene RG-3000 thermal cycler (Corbett Research, Australia). A three-step and a two-step cycling programme were investigated to ensure there were no potential differences in the results. Negative and positive controls were included in all runs. Two step cycling was carried out as follows: an initial step at 95°C for 10 min followed by 40 cycles of 95°C for 15 sec followed by 60°C for 60 sec. Three step cycling protocol is as follows: an initial step at 95°C for 10 min followed by 40 cycles of 95°C for 15 sec followed by 60°C for 60 sec and 72°C for 5 sec. Dissociation curves were generated at the end of each run for both two step and three step PCR programs, starting at 67°C and holding for 45 sec and then increasing 1 degree every 15 sec and finishing at 95°C. Cp values were calculated automatically by the Rotor-gene 3000 software. All qPCR reactions were replicated using an ABI7900HT Fast Real-Time PCR System (Applied Biosystems, USA) using a two step cycling program to ensure that the results obtained were independent of the thermal cycler platform used. All qPCR reaction products were subjected to gel (1.2% agarose) electrophoresis to verify amplification specificity.

## Supporting Information

Figure S1 **Gel electrophoresis products following gradient PCR of (A)** ***Ubi4*** **and (B)** ***Ubi10*** **using primers designed by Hong** ***et al.*** **(2008) (Ubi4FW, Ubi4RV and Ubi10Fw, Ubi10Rv)** [**5**] **, and primers designed to prime to the 3′-UTR (Ubi4-3Fw, Ubi4-3Rv and Ubi10-3Fw, Ubi10-3Rv).** Lanes 1 to 11: temperature gradient from 55 to 65°C at 1°C intervals, Lane 12: no RT control, Lane 13: water only blank, and Lane 14: PCR amplification at 60°C using primers targeting the 3′-UTR. Gel images are representative of 3 independent experiments.
(DOC)

Figure S2 **Binding sites of primers Ubi4Fw and Ubi4Rv on** ***Ubi4 and Ubi10*** **. The directions of priming are indicated by arrows. Mismatches are shown in red.**
(DOC)

Figure S3 **Binding sites of primers Ubi10Fw and Ubi10Rv on** ***Ubi10 and Ubi4*** **. The directions of priming are indicated by arrows. Mismatches are shown in red.**
(DOC)

Figure S4 **Comparison of the 3′-UTR regions of both** ***Ubi4*** **and** ***Ubi10*** **and binding sites of Ubi10-3Fw and Ubi10-3-Rv at the 3′-UTR of** ***Ubi10*** **, and Ubi4-3Fw and Ubi4-3Rv at the 3′-UTR of** ***Ubi4.***
(DOC)
