# Supplementary material for: Evaluation of the Use of the Polyubiquitin Genes, Ubi4 and Ubi10 as Reference Genes for Expression Studies in Brachypodium distachyon
Source: PLoS One. 2012 Nov 14;7(11):e49372. doi: 10.1371/journal.pone.0049372 (PMC3498167; doi:10.1371/journal.pone.0049372)
Supplement: Figure S1 — Gel electrophoresis products following gradient PCR of (A) Ubi4 and (B) Ubi10 using primers designed by Hong et al. (2008) (Ubi4FW, Ubi4RV and Ubi10Fw, Ubi10Rv) [5] , and primers designed to prime to the 3′-UTR (Ubi4-3Fw, Ubi4-3Rv and Ubi10-3Fw, Ubi10-3Rv). Lanes 1 to 11: temperature gradient from 55 to 65°C at 1°C intervals, Lane 12: no RT control, Lane 13: water only blank, and Lane 14: PCR amplification at 60°C using primers targeting the 3′-UTR. Gel images are representative of 3 independent experiments. (DOC) [file pone.0049372.s001.doc]

**Figure S1.**

Gel electrophoresis products following gradient PCR of (A) *Ubi4* and (B) *Ubi10* using primers designed by Hong *et al.* (2008) (Ubi4FW, Ubi4RV and Ubi10Fw, Ubi10Rv) [5], and primers designed to prime to the 3’-UTR (Ubi4-3Fw, Ubi4-3Rv and Ubi10-3Fw, Ubi10-3Rv). Lanes 1 to 11: temperature gradient from 55 to 65 °C at 1°C intervals, Lane 12: no RT control, Lane 13: water only blank, and Lane 14: PCR amplification at 60°C using primers targeting the 3’-UTR. Gel images are representative of 3 independent experiments.

**(A)**

Ubi4Fw and Rv, 55 °C

Ubi4Fw and Rv, 58°C

Ubi4Fw and Rv, 56 °C

Ubi4Fw and Rv, 60°C

Ubi4Fw and Rv, 59 °C

Ubi4Fw and Rv, 61°C

No RT Control

Ubi4Fw and Rv, 64 °C

Ubi4-3 Fw and Rv, 60 °C

Ubi4Fw and Rv, 63°C

Ubi4Fw and Rv, 62°C

Ubi4Fw and Rv, 65°C

Water

Ubi4Fw and Rv, 57°C

1

2

3

4

5

6

7

8

9

10

11

12

13

14


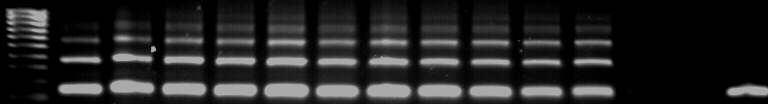


**(B)**

1

2

3

4

5

6

7

8

9

10

11

12

13

14

15


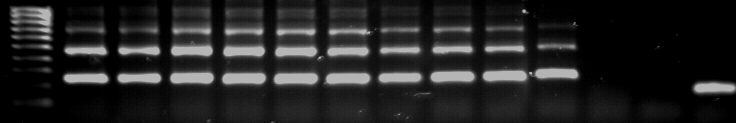


Ubi410w and Rv, 56 °C

Ubi10Fw and Rv, 59°C

Ubi10Fw and Rv, 58 °C

Ubi410Fw and Rv, 57 °C

Ubi10Fw and Rv, 62 °C

Ubi10Fw and Rv, 61°C

Ubi10Fw and Rv, 60 °C

Ubi10Fw and Rv, 64 °C

Ubi10Fw and Rv, 63 °C

No RT Control

Ubi10Fw and Rv, 65 °C

Ubi10-3 Fw and Rv, 60°C

Water
