# Supplementary material for: Evaluation of the Use of the Polyubiquitin Genes, Ubi4 and Ubi10 as Reference Genes for Expression Studies in Brachypodium distachyon
Source: PLoS One. 2012 Nov 14;7(11):e49372. doi: 10.1371/journal.pone.0049372 (PMC3498167; doi:10.1371/journal.pone.0049372)
Supplement: Figure S2 — Binding sites of primers Ubi4Fw and Ubi4Rv on Ubi4 and Ubi10 . The directions of priming are indicated by arrows. Mismatches are shown in red. (DOC) [file pone.0049372.s002.doc]

### Figure S2.

### Binding sites of primers Ubi4Fw and Ubi4Rv on *Ubi4 and Ubi10*. The directions of priming are indicated by arrows. Mismatches are shown in red.

**Binding sites of the primers Ubi4Fw and Ubi4Rv (Hong *et al.* (2008) [5]) on *Ubi4.* Mismatches are shown in red.**

C

ATGCAAATCTTTGTGAAGACTTTGACTGGCAAGACCATCACCCTTGAGGTGGAGTCATCTGACACCATCGACAATGTGAAGGCCAAGATCCAGGATAAGGAGGGCATCCCCCCTGACCAGCAGCGCCTTATCTTCGCAGGCAAGCAGCTTGAAGATGGCAGGACCCTTGCGGACTACAACATCCAGAAGGAGTCCACTCTGCACCTGGTCCTCCGTCTTCGGGGAGGCATGCAGATCTTCGTGAAGACACTGACAGGCAAGACCATCACCCTTGAGGTTGAGTCGTCAGACACCATTGACAATGTCAAGGCCAAGATCCAGGACAAGGAGGGCATCCCCCCGGACCAGCAGCGTTTGATCTTTGCTGGCAAGCAGCTGGAGGATGGTCGCACCCTTGCTGACTACAACATCCAGAAGGAGTCCACCCTTCACCTAGTGCTGCGTCTGCGTGGTGGTATGCAGATCTTTGTCAAGACCCTCACCGGCAAGACCATCACCTTGGAGGTTGAGAGCTCCGACACCATCGACAACGTGAAGGCCAAGATCCAGGACAAGGAGGGCATTCCCCCGGACCAGCAGCGTCTGATCTTCGCTGGCAAGCAGCTGGAGGATGGCCGCACCCTCGCTGACTACAACATCCAGAAGGAGTCCACCCTCCACCTTGTGCTCCGCCTTCGTGGTGGCATGCAGATCTTTGTCAAGACCCTCACCGGCAAGACCATCACCTTGGAGGTCGAGAGCTCCGACACCATCGACAACGTGAAGGCCAAGATCCAGGACAAGGAGGGCATTCCCCCGGACCAGCAGCGTCTGATCTTTGCAGGGAAG

T

T

C

CAGCTGGAGGATGGCCGCACCCTCGCTGACTACAACATCCAGAAGGAGTCCACCCTCCACCTTGTGCTCCGCCTTCGTGGTGGCATGCAGATCTTTGTCAAGACCCTCACCGGCAAGACCATCACCTTGGAGGTCGAGAGCTCTGACACCATCGACAACGTGAAGGCCAAGATCCAGGACAAGGAGGGCATTCCCCCGGACCAGCAGCGTCTGATCTTCGCAGGGAAGCAGCTGGAGGATGGCCGCACCCTCGCTGACTACAACATCCAGAAGGAGTCCACCCTCCACCTGGTGCTTCGTCTCCGTGGTGGTCAGTGA

**Binding sites of the primers Ubi10Fw and Ubi10Rv (Hong *et al.* (2008) [1]) on *Ubi4.* Mismatches are shown in red. Note the high degree of sequence overlap between Ubi10Fw and Ubi10Rv.**

ATGCAAATCTTTGTGAAGACTTTGACTGGCAAGACCATCACCCTTGAGGTGGAGTCATCTGACACCATCGACAATGTGAAGGCCAAGATCCAGGATAAGGAGGGCATCCCCCCTGACCAGCAGCGCCTTATCTTCGCAGGCAAGCAGCTTGAAGATGGCAGGACCCTTGCGGACTACAACATCCAGAAGGAGTCCACTCTGCACCTGGTCCTCCGTCTTCGGGGAGGCATGCAGATCTTCGTGAAGACACTGACAGGCAAGACCATCACCCTTGAGGTTGAGTCGTCAGACACCATTGACAATGTCAAGGCCAAGATCCAGGACAAGGAGGGCATCCCCCCGGACCAGCAGCGTTTGATCTTTGCTGGCAAGCAGCTGGAGGATGGTCGCACCCTTGCTGACTACAACATCCAGAAGGAGTCCACCCTTCACCTAGTGCTGCGTCTGCGTGGTGGTATGCAGATCTTTGTCAAGACCCTCACCGGCAAGACCATCACCTTGGAGGTTGAGAGCTCCGACACCATCGACAACGTGAAGGCCAAGATCCAGGACAAGGAGGGCATTCCCCCGGACCAGCAGCGTCTGATCTTCGCTGGCAAGCAGCTGGAGGATGGCCGCACCCTCGCTGACTACAACATCCAGAAGGAGTCCACCCTCCACCTT

A

C

T

G

C

A

A

A

T

G

GTGCTCCGCCTTCGTGGTGGCATGCAGATCTTTGTCAAGACCCTCACCGGCAAGACCATCACCTTGGAGGTCGAGAGCTCCGACACCATCGACAACGTGAAGGCCAAGATCCAGGACAAGGAGGGCATTCCCCCGGACCAGCAGCGTCTGATCTTTGCAGGGAAGCAGCTGGAGGATGGCCGCACCCTCGCTGACTACAACATCCAGAAGGAGTCCACCCTCCACCTTGTGCTCCGCCTTCGTGGTGGCATGCAGATCTTTGTCAAGACCCTCACCGGCAAGACCATCACCTTGGAGGTCGAGAGCTCTGACACCATCGACAACGTGAAGGCCAAGATCCAGGACAAGGAGGGCATTCCCCCGGACCAGCAGCGTCTGATCTTCGCAGGGAAGCAGCTGGAGGATGGCCGCACCCTCGCTGACTACAACATCCAGAAGGAGTCCACCCTCCACCTGGTGCTTCGTCTCCGTGGTGGTCAGTGA

A

T

G

A

A

T

A
