# Supplementary material for: Evaluation of the Use of the Polyubiquitin Genes, Ubi4 and Ubi10 as Reference Genes for Expression Studies in Brachypodium distachyon
Source: PLoS One. 2012 Nov 14;7(11):e49372. doi: 10.1371/journal.pone.0049372 (PMC3498167; doi:10.1371/journal.pone.0049372)
Supplement: Figure S4 — Comparison of the 3′-UTR regions of both Ubi4 and Ubi10 and binding sites of Ubi10-3Fw and Ubi10-3-Rv at the 3′-UTR of Ubi10 , and Ubi4-3Fw and Ubi4-3Rv at the 3′-UTR of Ubi4. (DOC) [file pone.0049372.s004.doc]

### Figure S4.

### Comparison of the 3’-UTR regions of both *Ubi4* and *Ubi10* and binding sites of Ubi10-3Fw and Ubi10-3-Rv at the 3’-UTR of *Ubi10*, and Ubi4-3Fw and Ubi4-3Rv at the 3’-UTR of *Ubi4.*

**Alignment of the 3’-UTRs of *Ubi4*  and *Ubi10.***

**3’-UTR of *Ubi10* showing the primer binding sites.**

GTGCCTGTCCATGGACTTGCTTCTGTCTGGGTTCACAAGTCTGGTTCAG

GGTGTCCTCCGGTGGAGTTGTCGCGTGTCTGAGTCTTATCTTTGATCTGA

TCGTGTGTTTGTTCCTACAGTACTGTCGTCAGTGTTATGCCTGTGTACCA

Ubi10-3Fw: 5’-TGGACTTGCTTCTGTCTGGGTTCA-3’

Ubi10-3Rv: 5’-TGGTACACAGGCATAACACTGACG-3’

**3’-UTR of *Ubi4* showing the primer binding sites.**

TGGCCTGCTGTTGGAACTGCTGCTATACCTGGGTCGTCGTCTGGTGGGT

GCCTGTGTGTTGCCCTTCATGAAGTGTGTCTCCGTGTTAAAGTCTTGTTG

AAGTCTACCTCTATCTGGTTAATGGACCATCGAGTCCCCTGGTGTGTGTT

GGTTTGGTGCAA

Ubi4-3Fw: 5’-GCTGTTGGAACTGCTGCTATACCT-3’

Ubi4-3Rv: 5’-TTGCACCAAACCAACACACACCAG-3’
